# Protective Effects of Coixol Against Nε-Carboxymethyllysine-Induced Injury in IMR-32 Neuronal Cells: Modulation of Endoplasmic Reticulum Stress and Amyloidogenic Pathways

**DOI:** 10.3390/nu17182939

**Published:** 2025-09-12

**Authors:** Mei-Chou Lai, Wayne Young Liu, Yu-Cheng Tzeng, I-Min Liu

**Affiliations:** 1 Department of Pharmacy and Master Program, Collage of Pharmacy and Health Care, Tajen University, Pingtung County 90741, Taiwan; 2 Department of Urology, Jen-Ai Hospital, Taichung City 412224, Taiwan; waynedoctor@gmail.com; 3 Collage of Nursing, Central Taiwan University of Science and Technology, Taichung City 40601, Taiwan; 4 Kaohsiung Medical University Hospital, Kaohsiung Medical University, Kaohsiung City 80708, Taiwan; hahahahanelson@gmail.com

**Keywords:** coixol, advanced glycation end products, Nε-carboxymethyllysine, endoplasmic reticulum stress, Aβ metabolism, IMR-32 cells

## Abstract

Background/Objectives: The accumulation of Nε-carboxymethyllysine (CML), a major advanced glycation end product (AGE), has been implicated in neuronal dysfunction by promoting oxidative stress, endoplasmic reticulum (ER) stress, and dysregulation of amyloid-β (Aβ) metabolism. This study evaluated the neuroprotective properties of coixol, a naturally occurring polyphenolic compound derived from the outer layers of *Coix lacryma-jobi* L. var. ma-yuen, in a CML-induced injury model using IMR-32 human neuronal-like cells. Methods: Cells were pretreated with coixol (1 μmol/L), *N*-acetyl-L-cysteine (NALC, 1 mmol/L), or 4-phenylbutyric acid (4-PBA, 200 μmol/L) for 1 h prior to CML (100 μmol/L) exposure for 24 h. Cell viability was determined by colorimetric analysis of 3-(4,5-dimethyl-2-yl)-2,5-diphenyltetrazolium bromide, while intracellular reactive oxygen species (ROS) generation was quantified using a fluorescence-based oxidative stress probe. Activities of key antioxidant enzymes and caspase-3 were determined using commercial assay kits. The expression of Aβ isoforms, amyloidogenic enzymes, ER stress markers, and apoptosis-related signaling proteins was quantified through validated immunoassays. Results: Coixol pretreatment significantly enhanced cell viability by attenuating ROS accumulation and restoring antioxidant enzyme activities. Concurrently, coixol suppressed ER stress signaling via downregulation of the protein kinase R-like ER kinase/C/EBP homologous protein axis and modulated apoptosis by increasing B-cell lymphoma (Bcl)-2, reducing Bcl-2-associated X protein expression, and inhibiting caspase-3 activation and DNA fragmentation. Furthermore, coixol regulated Aβ metabolism by inhibiting the expression of β-site amyloid precursor protein-cleaving enzyme 1 and presenilin 1, while restoring insulin-degrading enzyme and neprilysin levels, leading to reduced accumulation of Aβ40 and Aβ42. Conclusions: Compared to NALC and 4-PBA, coixol demonstrated comparable or superior modulation across multiple pathological pathways. These findings highlight coixol’s potential as a neuroprotective candidate in AGE-associated neurodegenerative conditions.

## 1. Introduction

Accumulating evidence indicates that individuals with impaired glucose tolerance or diabetes mellitus face a significantly elevated risk of developing neurodegenerative diseases, including but not limited to Alzheimer’s disease (AD), Parkinson’s disease, and vascular dementia [1]. These conditions are commonly characterized by progressive cognitive decline, memory impairment, motor dysfunction, and widespread neuronal loss, reflecting shared underlying mechanisms such as chronic oxidative stress, metabolic dysregulation, and impaired cellular resilience in the central nervous system [1]. Among the pathophysiological mechanisms underlying this increased susceptibility, the accumulation of advanced glycation end products (AGEs) and subsequent activation of their receptor RAGE have emerged as central contributors [2]. Activation of the AGEs–RAGE axis promotes sustained oxidative stress through enhanced reactive oxygen species (ROS) production and concurrently initiates endoplasmic reticulum (ER) stress responses, thereby amplifying neuronal dysfunction and damage [2,3].

Under chronic ER stress conditions, two main apoptotic pathways are triggered: one involves the activation of caspases through caspase-3 leading to programmed cell death; the other is mediated by the protein kinase-like endoplasmic reticulum kinase (PERK) pathway, where phosphorylation of eukaryotic initiation factor 2α (eIF2α) induces activating transcription factor 4 (ATF4) expression [4]. ATF4 subsequently induces the expression of C/EBP homologous protein (CHOP), a critical pro-apoptotic transcription factor that promotes cell death by suppressing the anti-apoptotic protein B-cell lymphoma 2 (Bcl-2) and increasing levels of pro-apoptotic proteins such as Bcl-2-associated X protein (Bax) [5]. Together, these ER-associated caspase and PERK/eIF2α/ATF4/CHOP signaling pathways act synergistically to drive neuronal apoptosis during prolonged ER stress [4]. Notably, in the context of diabetes, chronic ER stress has been closely linked to glucose-mediated neurotoxicity, contributing to impaired neuronal survival and function [3]. Moreover, prolonged ER stress may disrupt protein homeostasis and interfere with the degradation and clearance of neurotoxic peptides such as amyloid-beta (Aβ), potentially through the downregulation of Aβ-degrading enzyme [6]. These molecular events, collectively driven by AGE/RAGE-induced oxidative stress and ER dysfunction, highlight a critical pathological axis. As such, targeting this pathway may represent a viable therapeutic strategy to mitigate diabetes-associated neurodegenerative complications [7].

Coixol (6-methoxy-1,3-benzoxazol-2(3H)-one) is a bioactive polyphenolic compound primarily concentrated in the outer hull of adlay (*Coix lacryma-jobi* L. var. ma-yuen, commonly known as Job’s tears), a cereal grain extensively consumed throughout East Asia for both nutritional and medicinal purposes [8,9]. Previous studies have documented its broad pharmacological profile, including antioxidant, antimicrobial, anti-inflammatory, and melanogenesis-suppressing activities [10,11]. Furthermore, coixol has been shown to enhance glucose-stimulated insulin secretion via cAMP-dependent signaling, suggesting potential applications in glycemic control [12]. Owing to these diverse biological effects, coixol is considered a promising candidate for interventions targeting oxidative stress, metabolic dysregulation, and inflammatory pathologies. Recent evidence also indicates its neuroprotective potential, exemplified by its ability to attenuate Aβ_(25–35)_-induced cytotoxicity in PC12 neuronal model cells [13]. However, the translational relevance of these findings is limited using PC12 cells, which originate from rat pheochromocytoma and retain only partial neuronal properties, even after differentiation with nerve growth factor [14]. Moreover, earlier studies employed synthetic Aβ fragments as the primary neurotoxic insult [13], which do not adequately reflect the complex metabolic and oxidative stressors that contribute to neurodegeneration in chronic disease states such as diabetes. To address these limitations, the present study employs IMR-32 human neuroblastoma cells, which endogenously express key neurodegeneration-associated proteins, including RAGE and amyloid precursor protein (APP) [15,16], and respond robustly to stimulation by AGEs. In particular, Nε-carboxymethyllysine (CML), a well-characterized AGE and biomarker of glycation burden, has been implicated in both diabetic complications and neurodegenerative pathology through its interaction with RAGE, promoting oxidative stress, glial activation, and amyloid plaque stabilization [17,18]. By employing CML as the inducer of cellular stress, this study establishes a human-relevant in vitro model of AGEs–RAGE-mediated neurotoxicity.

Although coixol has been shown to inhibit Aβ aggregation and modulate acetylcholinesterase activity [13,19], its effects on AGEs-induced oxidative stress and Aβ clearance remain unclear. This study investigates coixol’s impact on the AGE–RAGE–mediated cascade leading to oxidative stress, ER stress, and disrupted Aβ metabolism. To benchmark its efficacy, two reference compounds were included as pharmacological comparators: *N*-acetyl-L-cysteine (NALC), an established ROS scavenger shown to inhibit ER stress under oxidative conditions [20], and 4-phenylbutyric acid (4-PBA), an FDA-approved chemical chaperone known to alleviate ER stress by stabilizing protein conformation and suppressing the unfolded protein response [21]. By integrating a pathophysiologically relevant stressor (CML), a human-derived neuronal model (IMR-32), and a comprehensive mechanistic framework, this study extends previous research and offers novel insights into the therapeutic potential of coixol in the context of AGEs-associated neurotoxicity.

## 2. Materials and Methods

### 2.1. Cells in Culture

IMR-32 cells (ATCC CCL-127) were sourced from the American Type Culture Collection (ATCC) and cultured in Eagle’s Minimum Essential Medium (EMEM, Cat#, M4655, Sigma-Aldrich, St. Louis, MO, USA) as specified by ATCC. The medium was supplemented with 10% fetal bovine serum (FBS; Cat# SH30070, Hyclone, Logan, UT, USA). Cells were maintained at 37 °C in a humidified incubator with 5% CO_2_ and grown in T-75 flasks at an initial seeding density of 3 × 10^5^ cells per flask. Subculturing was performed when cells reached approximately 75% confluency, using 0.25% trypsin–EDTA containing phenol red (Cat# 25200072, Gibco, Waltham, MA, USA) for detachment. To induce neuronal differentiation, IMR-32 cells were seeded at 1 × 10^5^ cells per T-25 flask and treated with all-trans retinoic acid (ATRA; Cat# R2625, Sigma-Aldrich, St. Louis, MO, USA), which was prepared as a 0.5 μg/μL stock solution in dimethyl sulfoxide (DMSO; Cat# 2650, Sigma-Aldrich, St. Louis, MO, USA) and diluted to a final concentration of 10 μmol/L in complete culture medium. Cells were exposed continuously to ATRA for 10 days, with medium replacement every 48 h. Upon completion of the 10-day differentiation period, cells were gently detached and re-plated into appropriate assay formats (6-well, 24-well, or 96-well plates) at densities optimized for each experiment. Replated cells were allowed to attach and recover for 24 h before further experimental treatments. This strategy ensured all assays were performed on IMR-32 cells at a consistent stage of neuronal differentiation, while allowing flexibility in cell density according to the specific assay requirements. Previous studies have demonstrated that ATRA-induced neuronal phenotypes in IMR-32 and other neuroblastoma cell models remain stable following gentle dissociation and replating, provided that appropriate recovery time and substrate coating are implemented [22].

### 2.2. CML Exposure and Pharmacological Interventions

Differentiated IMR-32 cells were re-plated into 6-well culture plates at a density of 2 × 10^6^ cells per well to provide sufficient cell numbers and total protein yield for subsequent biochemical analyses. Upon reaching the desired confluency, cells were detached using 0.05% (*w*/*v*) trypsin in phosphate-buffered saline (PBS, pH 7.4, Cat# P4474, Sigma-Aldrich, St. Louis, MO, USA). CML (≥95% purity, Cat# 16483, Cayman Chemical, Ann Arbor, MI, USA), 4-PBA (≥99% purity, Cat# P21005, Sigma-Aldrich, St. Louis, MO, USA), NALC (≥99% purity, Cat# A9165, Sigma-Aldrich, St. Louis, MO, USA), and coixol (≥97% purity, Cat# 543551, Sigma-Aldrich, St. Louis, MO, USA) were prepared as stock solutions as follows: CML, 100 mmol/L in PBS; NALC, 100 mmol/L in PBS; coixol, 10 mmol/L in DMSO; and 4-PBA, 1 mol/L in DMSO. These stock concentrations were selected to facilitate preparation of the desired working solutions while ensuring that the final DMSO concentration in the culture medium did not exceed 0.1% (*v*/*v*), a level previously reported to be non-cytotoxic [23]. Vehicle controls consisted of culture medium containing ≤0.1% (*v*/*v*) DMSO, prepared identically to the treatment solutions but without the active compounds.

To determine cytotoxic concentrations, cells were exposed to CML at 25, 50, 100, or 200 μmol/L and to coixol at 0.25, 0.5, 1, or 2 μmol/L for 24 h. Based on cell viability results, 100 μmol/L CML, which reduced cell viability to approximately 50%, was selected for subsequent experiments. To evaluate the cytoprotective potential of coixol, IMR-32 neuroblastoma cells were pretreated for 1 h with increasing concentrations of coixol (0.25, 0.5, 1, or 2 μmol/L), as previously reported [13]. For comparative purposes, two established pharmacological agents were employed as positive controls: NALC (1 mmol/L) [24] and 4-PBA (200 μmol/L) [25]. Following pretreatment, all experimental groups were exposed to 100 μmol/L CML for 24 h at 37 °C to induce neurotoxic stress.

### 2.3. Cytotoxicity Evaluation

For cytotoxicity assays, differentiated IMR-32 cells were re-plated into 24-well plates. This format was chosen to optimize reagent volumes and formazan solubilization efficiency while maintaining consistent cell monolayers for accurate optical density measurements. Cells were incubated with 3-(4,5-dimethylthiazol-2-yl)-2,5-diphenyltetrazolium bromide (MTT; Cat# M2003, Sigma-Aldrich, St. Louis, MO, USA) for 2 h to assess cell viability, as previously described [26]. Cell morphology was concurrently observed under a phase contrast microscopy (IX71; Olympus Corporation, Tokyo, Japan). Metabolically active cells reduced the yellow MTT reagent to insoluble purple formazan crystals, which were subsequently dissolved in acidified isopropanol. The absorbance of the resulting solution was measured at 570 nm using a microplate spectrophotometer (SpectraMax M5; Molecular Devices, Sunnyvale, CA, USA). The amount of formazan produced was considered directly proportional to the number of viable cells. Cell viability was expressed as a percentage relative to untreated control cells, which were set at 100%.

### 2.4. Measurement of Oxidative Stress Markers

Differentiated IMR-32 cells were harvested from T-25 flasks after completion of the 10-day ATRA-induced differentiation regimen and re-plated into 96-well plates at a density of 1 × 10^5^ cells per well for subsequent treatment with vehicle (control), coixol (1 μmol/L), NALC (1 mmol/L), or 4-PBA (200 μmol/L). This format was chosen to enable high-throughput microplate-based measurements and ensure uniform cell layers for reproducible fluorescence and absorbance readings. Although the density was relatively high, it was applied only for short-term incubations (≤24 h) in 200 μL of complete medium per well, minimizing the risk of nutrient depletion or metabolic stress. Morphology and medium pH (via phenol red) were monitored before measurements, and no signs of nutrient exhaustion, acidification, or spontaneous detachment were observed in vehicle-treated controls.

After a 1 h pretreatment, 100 μmol/L of CML was added, and cells were incubated for an additional 24 h at 37 °C. Intracellular ROS levels were assessed using the redox-sensitive fluorescent dye 2′,7′-dichlorodihydrofluorescein diacetate (DCFH-DA), which undergoes intracellular deacetylation followed by oxidation to yield fluorescent dichlorofluorescein (DCF) in the presence of peroxides [27]. Following treatment, the culture medium was removed and replaced with fresh medium containing 5 μg/mL DCFH-DA (Cat# 35845, Sigma-Aldrich, St. Louis, MO, USA). Cells were incubated with the probe for 30 min at 37 °C. Representative fluorescence images were obtained using a fluorescence microscope (Leica Microsystems, Wetzlar, Hessen, Germany). Fluorescence intensity was continuously measured for 30 min using a microplate reader (SpectraMax M5; Molecular Devices, Sunnyvale, CA, USA) at excitation and emission wavelengths of 488 nm and 525 nm, respectively. Quantification was performed on images captured from six randomly selected, non-overlapping fields per well in three independent experiments, resulting in a total of 18 fields per treatment group. The random and non-overlapping field selection across replicate wells was intended to minimize the potential influence of cell density variations. The level of ROS was expressed as a percentage relative to the untreated control group for each experimental replicate.

The enzymatic activities of superoxide dismutase (SOD; Cat# ab65354; Abcam, Cambridge, UK), catalase (CAT; Cat# ab83464; Abcam, Cambridge, UK), glutathione peroxidase (GPx; Cat# ab102530; Abcam, Cambridge, UK), and glutathione reductase (GR; Cat# ab83461; Abcam, Cambridge, UK) were quantified using commercially available assay kits, following the manufacturer’s protocols. SOD activity was evaluated by measuring absorbance at 450 nm, while CAT, GPx, and GR activities were assessed by monitoring absorbance at 570 nm, 340 nm, and 405 nm, respectively, using a microplate reader (SpectraMax M5; Molecular Devices, Sunnyvale, CA, USA). All enzymatic activities were normalized to total protein concentration, determined via the bicinchoninic acid (BCA) protein assay (Cat# ab102536; Abcam, Cambridge, UK).

### 2.5. Quantification of Aβ Species and Aβ-Related Enzymes

IMR-32 cells were seeded in 96-well plates at a density of 1 × 10^5^ cells per well and subsequently treated with vehicle (control), coixol (1 μmol/L), NALC (1 mmol/L), or 4-PBA (200 μmol/L). Following a 1 h pretreatment, CML (100 μmol/L) was added, and cells were incubated for an additional 24 h at 37 °C. To assess Aβ-related secretory profiles, the culture supernatants were harvested and centrifuged at 12,000 rpm for 15 min at 4 °C to eliminate cellular debris. The concentrations of Aβ40 and Aβ42 in the clarified supernatants were quantified using sandwich ELISA kits (Aβ40: Cat# abx255205; Aβ42: Cat# abx255206; Abbexa Ltd., Cambridge, UK) according to the manufacturer’s instructions.

In addition, the levels of β-site amyloid precursor protein-cleaving enzyme 1 (BACE1), presenilin 1 (PS1), insulin-degrading enzyme (IDE), and neprilysin (NEP) were measured using specific ELISA kits (BACE1: Cat# abx574903; PS1: Cat# abx350520; IDE: Cat# abx154173; NEP: Cat# abx154443; Abbexa Ltd., Cambridge, UK). Absorbance was recorded at 450 nm using a microplate reader (SpectraMax M5, Molecular Devices, Sunnyvale, CA, USA). All data were normalized to total protein concentration, determined via the BCA protein assay (Cat# ab102536; Abcam, Cambridge, UK).

### 2.6. Qualitative Assessment of ER Stress Markers and Bcl-2 Family Proteins

To assess ER stress responses, IMR-32 cells were seeded in 96-well plates at a density of 1 × 10^5^ cells per well and pretreated with either vehicle (control), coixol (1 μmol/L), NALC (1 mmol/L), or 4-PBA (200 μmol/L) for 1 h at 37 °C. Subsequently, cells were exposed to 100 μmol/L CML and incubated for an additional 24 h under identical conditions. Following treatment, cells were fixed in situ for the qualitative assessment of ER stress-associated markers using commercially available ELISA kits. Phosphorylated PERK (Thr981) and total PERK were quantified using colorimetric ELISA kits (Cat# CBP2027 and CB5548; Assay Biotechnology Inc., San Jose, CA, USA). Total eukaryotic initiation factor 2 alpha (eIF2α) and its phosphorylated form (Ser51) were measured with corresponding kits (Cat# CB5226 and CBP1538; Assay Biotechnology Inc., San Jose, CA, USA). Activating transcription factor 4 (ATF4) expression was analyzed using a colorimetric ELISA kit (Cat# TE-0039; Signosis, Santa Clara, CA, USA), and C/EBP homologous protein (CHOP) levels were determined via a sandwich ELISA protocol (Cat# EM1933; Fine Biotech Co., Ltd., Wuhan, China). To evaluate apoptosis-related protein expression, B-cell lymphoma 2 (Bcl-2) and Bcl-2-associated X protein (Bax) levels were determined using ELISA kits (Cat# CBCAB00158 and CBCAB00157; Assay Genie, Dublin, Ireland). All assays were performed in accordance with the manufacturers’ protocols. Briefly, cells were fixed and permeabilized, followed by blocking with assay buffer for 1 h at room temperature. Primary antibody incubation was carried out for 2 h, followed by a 1 h incubation with horseradish peroxidase-conjugated secondary antibodies. The colorimetric reaction was developed by adding a substrate solution and incubating in the dark for 10–20 min at room temperature. Absorbance was measured at 450 nm using a microplate reader (SpectraMax M5, Molecular Devices, Sunnyvale, CA, USA). Protein expression levels were normalized to total protein content, determined using a BCA protein assay (Cat# ab102536; Abcam, Cambridge, UK), and expressed relative to the untreated control group.

### 2.7. Quantification of Caspase-3-like Enzymatic Activity

Caspase-3-like enzymatic activity was assessed using a colorimetric assay kit (Cat# ab39401; Abcam, Cambridge, UK) following the manufacturer’s instructions. Cellular extracts containing 200 μg of total protein, quantified using the BCA protein assay, were incubated with 200 μmol/L of the caspase-specific substrate Ac-DEVD-pNA in reaction buffer at 37 °C for 2 h. The cleavage of Ac-DEVD-pNA by activated caspase-3-like proteases released pNA, which was quantified by measuring absorbance at 405 nm using a SpectraMax M5 microplate reader (Molecular Devices, USA). All measurements were normalized to the values obtained from the untreated control group.

### 2.8. ELISA-Based Quantification of DNA Fragmentation

DNA fragmentation was evaluated using a Cell Death Detection ELISA kit (Cat# 11544675001; Roche Molecular Biochemicals, Mannheim, Germany), which quantitatively detects histone-associated DNA fragments released into the cytoplasm during apoptotic cell death. Cytoplasmic extracts were prepared according to the manufacturer’s instructions and used as antigen sources in a sandwich ELISA format. Briefly, microtiter plates pre-coated with a mouse monoclonal anti-histone antibody were employed to capture nucleosome-bound histones present in the cytoplasmic fractions. Subsequently, a peroxidase-conjugated mouse monoclonal anti-DNA antibody was added to form histone–DNA immunocomplexes. Enzymatic activity was visualized using 2,2′-azino-bis(3-ethylbenzothiazoline-6-sulfonic acid) as the chromogenic substrate. The reaction was carried out at 20 °C for 10 min in the dark. The extent of DNA fragmentation was determined by measuring the absorbance at 405 nm using a microplate reader (SpectraMax M5; Molecular Devices, Sunnyvale, CA, USA).

### 2.9. Statistical Analysis

All data are presented as mean ± standard deviation (SD) from five independent experiments (*n* = 5), with each experimental group measured in technical triplicate wells. Statistical analyses were performed using one-way analysis of variance (ANOVA) followed by Tukey’s post hoc test for multiple comparisons (SigmaPlot, version 14; Systat Software, Inc., San Jose, CA, USA). A *p*-value < 0.05 was considered statistically significant.

## 3. Results

### 3.1. Coixol Attenuates CML-Induced Cytotoxicity

**Figure 1:**
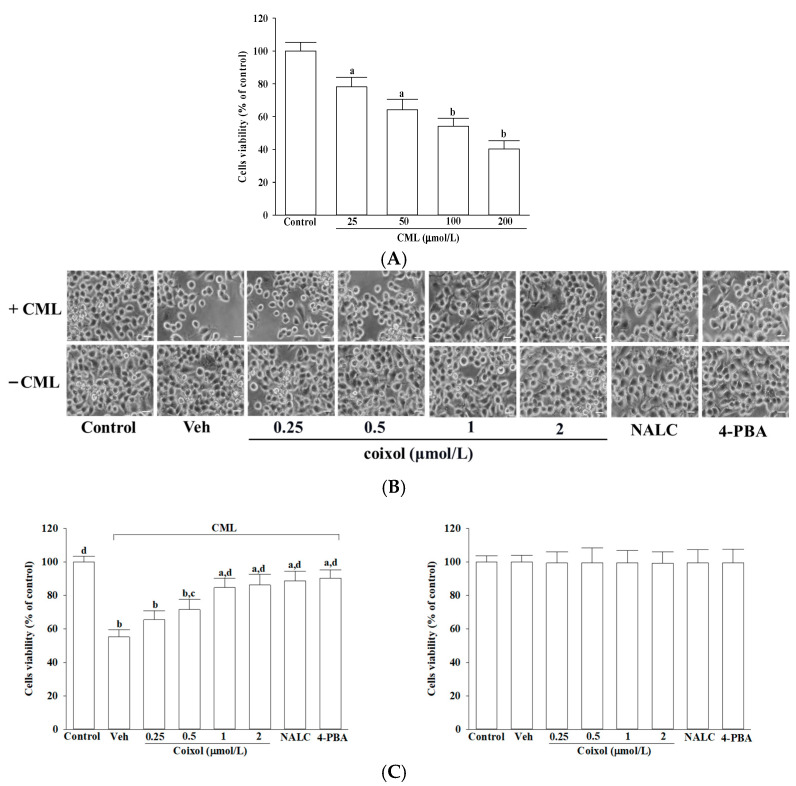
Effects of treatments on the cell viability in CML-treated IMR-32 cells. (**A**) IMR-32 cells were exposed to increasing concentrations of CML for 24 h to evaluate concentration-dependent cytotoxicity. (**B**) Cells were pretreated with coixol (0.25, 0.5, 1, or 2 μmol/L), NALC (1 mmol/L), or 4-PBA (200 μmol/L) for 1 h, followed by incubation with (+) or without (−) 100 μmol/L CML for an additional 24 h. Representative cell morphology images were captured by phase contrast microscopy at 100× original magnification; scale bar: 100 μm (**upper panel**). (**C**) Cell viability was quantified by MTT assay and expressed as a percentage relative to the untreated control group (**lower panel**). The results are presented as the mean ± SD of five independent experiments (*n* = 5), each conducted in triplicate. ^a^ *p* < 0.05 and ^b^ *p* < 0.01 compared to the data from untreated control group (control). ^c^ *p* < 0.05 and ^d^ *p* < 0.01 when compared to the data from vehicle (Veh)-treated cells exposed to CML.

To establish a neuronal injury model, IMR-32 cells were exposed to increasing concentrations of CML (25, 50, 100, or 200 μmol/L) for 24 h, which resulted in a concentration-dependent reduction in cell viability (Figure 1A). Among the tested concentrations, 100 μmol/L CML, which reduced cell viability to approximately 54.1% (*p* < 0.001 vs. control), was selected for subsequent experiments, as higher concentrations such as 200 μmol/L further decreased viability to around 40.2% (*p* < 0.001 vs. control; Figure 1A).

As shown in the microscopy images (Figure 1B), exposure of differentiated IMR-32 cells to CML resulted in marked morphological deterioration, characterized by soma shrinkage, loss of neurite extensions, and reduced phase-bright appearance. Coixol pretreatment preserved morphology in a concentration-dependent manner. Quantitative analysis further confirmed these observations (Figure 1C). Pretreatment with 1 μmol/L coixol increased the survival rate of CML-treated IMR-32 cells to 84.6% (*p* < 0.001 vs. CML), while 2 μmol/L coixol pretreatment resulted in a similar survival rate of 86.2% (*p* < 0.001 vs. CML), with no significant difference between the two concentrations (*p* = 0.68). Accordingly, 1 μmol/L coixol was selected for further investigations. Pretreatment with NALC (1 mmol/L) or 4-PBA (200 μmol/L) also conferred significant cytoprotective effects, restoring cell viability to 88.6% and 93.3%, respectively (*p* < 0.001 vs. CML). Comparative analysis showed that the protective effect of coixol was statistically comparable to NALC (*p* = 0.21 vs. coixol) and 4-PBA (*p* = 0.09 vs. coixol). Treatment with coixol, NALC, or 4-PBA alone did not significantly affect basal cell viability (*p* > 0.05 vs. control), indicating that the observed protective effects were specific to CML-induced stress.

### 3.2. Coixol Attenuates CML-Induced Oxidative Stress and Antioxidant Dysfunction in IMR-32 Cells

**Figure 2:**
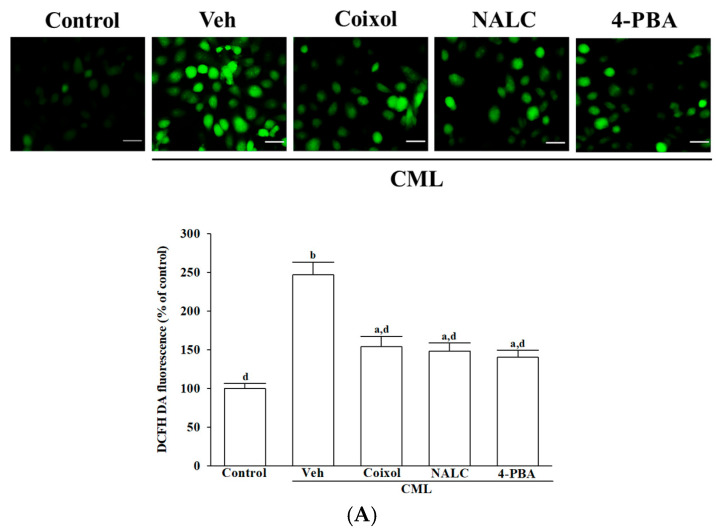

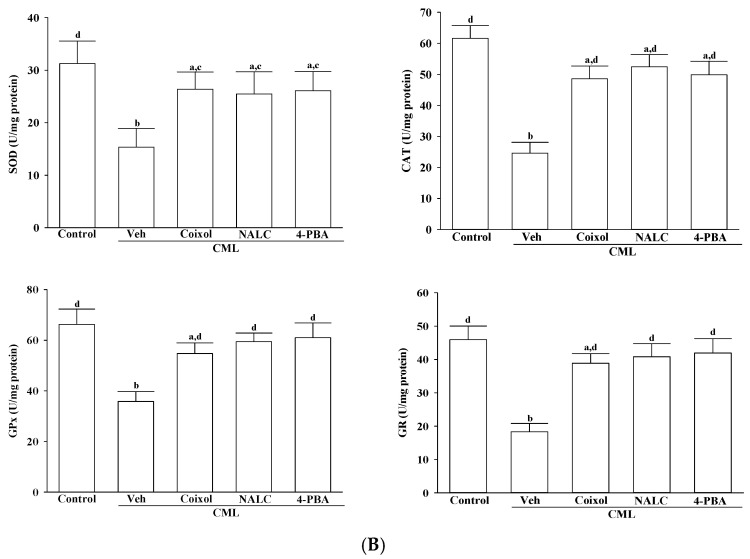
Effects of treatments on intracellular ROS production and antioxidant enzyme activities in CML-treated IMR-32 cells. Cells were pretreated with coixol (1 μmol/L), NALC (1 mmol/L) or 4-PBA (200 μmol/L) for 1 h, followed by stimulation with CML (100 μmol/L) for 24 h. (**A**) Representative fluorescence images (**upper panel**) illustrate intracellular ROS levels detected by DCFH-DA staining. Original magnification:100×; scale bar: 100 μm. Quantitative analysis of fluorescence intensity (**lower panel**) was normalized to total protein content, with the untreated control group set at 100% as the reference. (**B**) Activities of SOD, CAT, GPx, and GR were assessed. The results are presented as the mean ± SD of five independent experiments (*n* = 5), each conducted in triplicate. ^a^ *p* < 0.05 and ^b^ *p* < 0.01 compared to the data from untreated control group (control). ^c^ *p* < 0.05 and ^d^ *p* < 0.01 when compared to the data from vehicle (Veh)-treated cells exposed to CML.

Intracellular ROS production, as indicated by DCFH-DA fluorescence, was visualized using a fluorescence microscope (Figure 2A, upper panel). CML increased intracellular ROS by 2.5-fold (*p* < 0.001 vs. control, Figure 2A). Coixol reduced ROS by 37.6% (*p* < 0.001 vs. CML), comparable to NALC (39.8%, *p* = 0.42 vs. coixol) and 4-PBA (42.3%, *p* = 0.36 vs. coixol; Figure 2A, lower panel).

CML reduced SOD activity by 50.2% (*p* < 0.001 vs. control). Coixol restored SOD to 1.7-fold of the CML group (*p* < 0.001 vs. CML), comparable to NALC (*p* = 0.48 vs. coixol) and 4-PBA (*p* = 0.95 vs. coixol). Similar trends were observed for CAT, GPx, and GR activities, with all three agents showing comparable effects (*p* > 0.05 for all coixol vs. NALC/4-PBA comparisons; Figure 2B).

### 3.3. Coixol Restores Aβ Homeostasis in CML-Treated IMR-32 Cells

**Figure 3:**
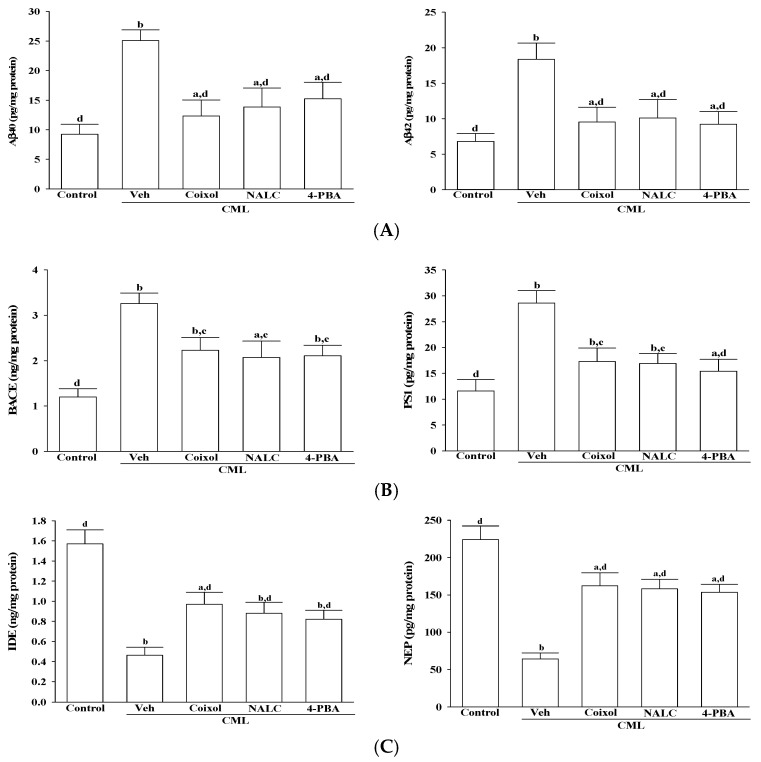
Effects of treatments on Aβ species and Aβ-related enzymes in CML-treated IMR-32 cells. IMR-32 cells were pretreated with coixol (1 μmol/L), NALC (1 mmol/L) or 4-PBA (200 μmol/L) for 1 h, followed by exposure to CML (100 μmol/L) for 24 h. (**A**) The protein levels of Aβ species, including Aβ40 and Aβ42, were quantified. (**B**) Levels of BACE1 and PS1 were measured to assess amyloidogenic processing. (**C**) The expression of Aβ-degrading enzymes, including IDE and NEP, was qualitatively evaluated. The results are presented as the mean ± SD of five independent experiments (*n* = 5), each conducted in triplicate. ^a^ *p* < 0.05 and ^b^ *p* < 0.01 compared to the data from untreated control group (control). ^c^ *p* < 0.05 and ^d^ *p* < 0.01 when compared to the data from vehicle (Veh)-treated cells exposed to CML.

Exposure to CML increased intracellular Aβ levels in IMR-32 cells, with Aβ40 and Aβ42 levels rising by 2.7-fold and 2.6-fold, respectively, compared with the untreated control group (*p* < 0.001 vs. control; Figure 3A). Pretreatment with coixol (1 μmol/L) suppressed these elevations, reducing Aβ40 and Aβ42 to 49.1% and 51.8% of the CML-treated group (*p* < 0.001 vs. CML), respectively. Similar reductions were observed following pretreatment with NALC (1 mmol/L), which lowered Aβ40 and Aβ42 by 44.7% and 44.9% (*p* = 0.34 and 0.41 vs. coixol), respectively, and with 4-PBA (200 μmol/L), which reduced these peptides by 39.3% and 49.8% (*p* = 0.28 and 0.39 vs. coixol), respectively (Figure 3A).

Moreover, CML stimulation increased the expression of BACE1 and PS1 (Figure 3B). Coixol pretreatment effectively attenuated this upregulation, decreasing BACE1 and PS1 protein levels by 31.6% and 39.6%, respectively, relative to the CML group. NALC pretreatment resulted in similar suppressive effects, reducing BACE1 and PS1 by 36.5% and 40.9% (*p* = 0.49 and 0.58 vs. coixol), respectively. Likewise, 4-PBA pretreatment led to reductions of 30.1% and 44.1% for BACE1 and PS1 (*p* = 0.63 and 0.47 vs. coixol), respectively (Figure 3B).

CML treatment suppressed the expression of IDE and NEP, reducing their levels to 33.2% and 35.1% of control levels, respectively (Figure 3C). Pretreatment with coixol restored IDE and NEP expression to 63.9% and 72.3% of control (*p* < 0.001 vs. CML), comparable to NALC and 4-PBA (*p* > 0.05 for all comparisons; Figure 3C).

### 3.4. Coixol Alleviates CML-Induced Endoplasmic Reticulum Stress in IMR-32 Cells

**Figure 4:**
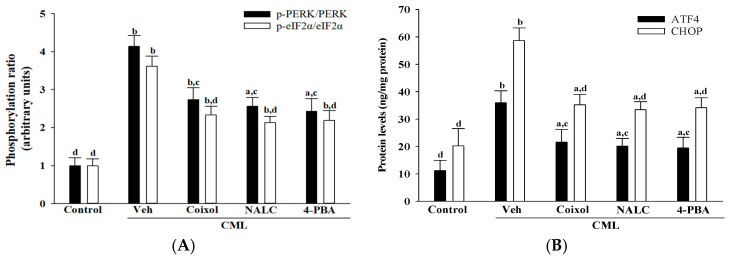
Effects of treatments on the ER stress molecules in CML-treated IMR-32 cells. Cells were pretreated with coixol (1 μmol/L), NALC (1 mmol/L) or 4-PBA (200 μmol/L) for 1 h prior to exposure to CML (100 μmol/L) for 24 h. (**A**) Protein expression levels of phosphorylated PERK (*p*-PERK) and eIF2α (*p*-eIF2α) were assessed, and the ratios of *p*-PERK/PERK and *p*-eIF2α/eIF2α were calculated. (**B**) Expression levels of downstream ER stress markers ATF4 and CHOP were determined. The results are presented as the mean ± SD of five independent experiments (*n* = 5), each conducted in triplicate. ^a^ *p* < 0.05 and ^b^ *p* < 0.01 compared to the data from untreated control group (control). ^c^ *p* < 0.05 and ^d^ *p* < 0.01 when compared to the data from vehicle (Veh)-treated cells exposed to CML.

CML exposure markedly elevated the *p*-PERK/PERK and *p*-eIF2α/eIF2α ratios by 4.1-fold and 3.6-fold, respectively (*p* < 0.001 vs. control; Figure 4A). Coixol pretreatment attenuated these increases by 33.8% and 35.6% (*p* < 0.001 vs. CML). The reduction in the *p*-PERK/PERK ratio was statistically comparable to that achieved by 4-PBA (*p* = 0.29 vs. coixol) but significantly greater than that of NALC (*p* = 0.017 vs. coixol). For the *p*-eIF2α/eIF2α ratio, all three treatments produced comparable effects (*p* > 0.05).

Similarly, CML treatment increased ATF4 and CHOP protein levels by 3.2-fold and 2.9-fold, respectively (*p* < 0.001 vs. control; Figure 4B). Coixol reduced these elevations by 39.9% and 40.1% (*p* < 0.001 vs. CML), with no significant differences relative to NALC or 4-PBA (*p* > 0.05 for all comparisons; Figure 4B).

### 3.5. Coixol Alleviates CML-Induced ER Stress-Mediated Cell Death in IMR-32 Cells

**Figure 5:**
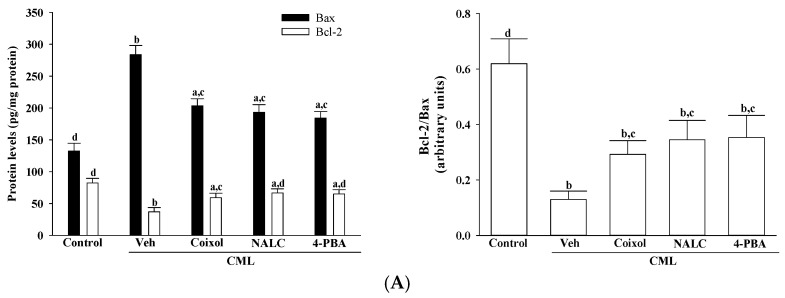

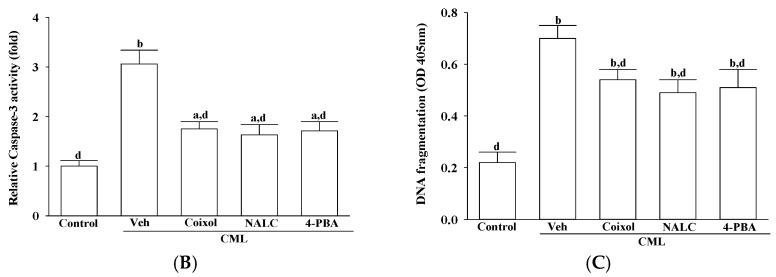
Effects of treatments on ER stress-associated cell death in CML-treated IMR-32 cells. Cells were pretreated with coixol (1 μmol/L), NALC (1 mmol/L) or 4-PBA (200 μmol/L) for 1 h, followed by exposure to CML (100 μmol/L) for 24 h. (**A**) Protein expression levels of Bax and Bcl-2 were determined, and the Bcl-2/Bax ratio was calculated. (**B**) Caspase-3-like enzymatic activity was measured. (**C**) DNA fragmentation was quantified using a cell death detection ELISA. The results are presented as the mean ± SD of five independent experiments (*n* = 5), each conducted in triplicate. ^a^ *p* < 0.05 and ^b^ *p* < 0.01 compared to the data from untreated control group (control). ^c^ *p* < 0.05 and ^d^ *p* < 0.01 when compared to the data from vehicle (Veh)-treated cells exposed to CML.

CML exposure markedly increased Bax expression and decreased Bcl-2 expression (*p* < 0.001 vs. control), resulting in a reduced Bcl-2/Bax ratio. Pretreatment with coixol restored the Bcl-2/Bax ratio by 2.2-fold (*p* < 0.001 vs. CML), an effect that was statistically comparable to that of NALC (*p* = 0.18 vs. coixol) and 4-PBA (*p* = 0.14 vs. coixol; Figure 5A).

Similarly, CML increased caspase-3 activity by approximately 3-fold (*p* < 0.001 vs. control). Coixol pretreatment reduced caspase-3 activity by 42.8% (*p* < 0.001 vs. CML), with no significant difference compared to NALC (*p* = 0.22 vs. coixol) or 4-PBA (*p* = 0.30 vs. coixol; Figure 5B).

In addition, CML induced a ~3.1-fold increase in DNA fragmentation (*p* < 0.001 vs. control). Coixol reduced DNA fragmentation by 23.8% (*p* < 0.001 vs. CML), an effect that was comparable to those observed with NALC (*p* = 0.37 vs. coixol) and 4-PBA (*p* = 0.41 vs. coixol; Figure 5C).

## 4. Discussion

The present study demonstrates that coixol, a key polyphenolic constituent of adlay, exerts neuroprotective effects in IMR-32 human neuroblastoma cells subjected to CML-induced toxicity. Rather than relying on exogenous Aβ fragments to model AD–like pathology, our study employs CML, a clinically relevant AGEs to activate endogenous RAGE signaling [28]. This human cell–based model better recapitulates the chronic metabolic and proteotoxic stress observed in diabetes-associated neurodegeneration. Under these conditions, coixol markedly improved neuronal resilience, primarily through suppression of ER stress and normalization of Aβ metabolism. These findings suggest that coixol may offer therapeutic potential in targeting the AGE/RAGE axis in neurodegenerative diseases.

AGEs such as CML are recognized contributors to AD pathology, largely through activation of the RAGE receptor, which promotes oxidative stress, ER dysfunction, and Aβ aggregation [17,28]. In our experimental model, CML exposure led to cytotoxicity and elevated intracellular ROS levels in human neuronal cells, consistent with prior studies in neuronal systems [4]. Coixol pretreatment effectively counteracted CML-induced oxidative stress by reducing intracellular ROS accumulation and preserving antioxidant enzyme activities, including SOD, CAT, GPx, and GR. These enzymes are critical for maintaining neuronal redox homeostasis, particularly given the vulnerability of neurons to oxidative insults due to their high energy demands and limited repair capacity [29]. Our results extend previous findings in non-neuronal systems [10,11] and establish coixol’s antioxidant and cytoprotective efficacy in a disease-relevant human neuronal context, underscoring its translational potential in AGEs-related neurodegeneration.

Coixol restored the CML-disrupted redox homeostasis, exhibiting antioxidant capacity comparable to NALC, a prototypical ROS scavenger, and 4-PBA, a chemical chaperone known to alleviate ER stress [24,25]. This dual action underscores coixol’s broad cytoprotective profile, as it not only suppresses oxidative stress but also attenuates downstream ER stress responses. In particular, coixol markedly inhibited the activation of the PERK/eIF2α/ATF4/CHOP axis, a canonical ER stress signaling pathway implicated in apoptosis [5]. The observed reductions in PERK and eIF2α phosphorylation, coupled with diminished ATF4 and CHOP expression, were comparable to those elicited by 4-PBA, suggesting that coixol may act through chaperone-like mechanisms or upstream regulatory modulation.

Coixol-mediated attenuation of ER stress was closely associated with a significant reduction in apoptosis-related signaling, underscoring the mechanistic interplay between unresolved ER stress and intrinsic apoptotic activation [30]. Coixol upregulated Bcl-2 while downregulating Bax, restoring the Bcl-2/Bax ratio-a critical determinant of mitochondrial membrane integrity and cell survival [31]. Additionally, coixol suppressed caspase-3 activity and DNA fragmentation, key indicators of apoptotic execution [32,33]. These effects align with the pro-apoptotic role of CHOP, which, upon activation via the PERK/eIF2α/ATF4 axis, represses Bcl-2 and promotes Bax and caspase-3 expression [4,34]. By concurrently modulating CHOP and its downstream effectors, coixol interrupts multiple apoptotic checkpoints downstream of ER stress. These findings support coixol’s capacity to mitigate AGE-induced neurotoxicity through coordinated anti-apoptotic and ER stress-relieving mechanisms.

In addition to alleviating oxidative and ER stress, coixol exhibited a marked regulatory effect on Aβ metabolism, a core pathological hallmark of AD. Specifically, coixol markedly reduced CML-induced elevations of Aβ40 and Aβ42, key isoforms involved in neurotoxicity, Aβ40 in vascular deposition and Aβ42 in plaque formation, both of which impair synaptic function and plasticity [35]. Thus, the ability of coixol to concurrently suppress the accumulation of Aβ40 and Aβ42 underscores its therapeutic potential in mitigating amyloid-driven neurodegeneration. The aberrant accumulation of Aβ is largely attributed to increased amyloidogenic processing of APP by BACE1 and PS1, a core component of the γ-secretase complex [36]. In our model, coixol significantly reduced CML-induced upregulation of both BACE1 and PS1, indicating a potential capacity to limit Aβ production at its enzymatic source. Concurrently, coixol restored the expression of IDE and NEP, two critical proteases responsible for Aβ clearance [37]. Given that chronic ER stress has been reported to suppress IDE and NEP expression, thereby aggravating Aβ burden and accelerating disease progression [6], our findings suggest that coixol may counteract this pathogenic cascade by targeting both production and clearance mechanisms. While previous studies on coixol have primarily focused on its antioxidative or anti-inflammatory properties in non-neuronal or systemic models [9,10], our results expand its functional profile by revealing its dual regulatory role in Aβ metabolism within human neuronal cells. Importantly, this is the first study to show that coixol can simultaneously downregulate amyloidogenic enzymes and restore Aβ-degrading proteases, supporting its potential as a neuroprotective candidate. Nevertheless, further investigations are needed to elucidate the upstream molecular pathways by which coixol modulates BACE1 and PS1 expression, to explore other therapeutic targets for Aβ metabolism such as α-secretase activation or microglial-mediated Aβ clearance, and to assess the activity of structurally related coixol derivatives with potentially enhanced pharmacological properties.

Coixol exhibited a broad and integrative cytoprotective profile by concurrently targeting oxidative stress, ER stress, and Aβ metabolism—three interrelated pathological processes in AD. Like NALC, coixol effectively reduced intracellular ROS levels and restored the activity of key antioxidant enzymes [20]. Similarly, its effects on ER stress paralleled those of 4-PBA, as evidenced by the suppression of PERK and eIF2α phosphorylation and downregulation of downstream ATF4 and CHOP expression [21]. Coixol also modulated Aβ homeostasis by reducing the expression of BACE1 and PS1, key enzymes involved in amyloidogenic APP processing, while enhancing the expression of the Aβ-degrading enzymes IDE and NEP. These changes were associated with reductions in both Aβ40 and Aβ42 levels, with effects comparable to those observed for NALC and 4-PBA under the present experimental conditions. As a bioactive compound derived from a traditionally consumed plant, coixol may offer advantages in safety and availability over synthetic agents [9]. Its long-standing inclusion in the human diet supports the potential for good biocompatibility, low toxicity, and suitability for long-term use. These attributes, combined with its multitarget activity profile, suggest that coixol remains a promising neuroprotective candidate with potential translational value, while future studies should include dose–response and head-to-head comparisons with reference compounds to clarify its relative pharmacological profile.

This study presents the first experimental evidence that coixol modulates a sequential neurodegenerative cascade initiated by AGE–RAGE interaction, which triggers ROS generation, ER stress activation, and aberrant Aβ metabolism in human neuronal cells. By employing differentiated IMR-32 cells—known to endogenously express key Alzheimer’s disease–related proteins—we established a human-relevant model that strengthens the translational significance of our findings. Within this system, coixol not only restored redox homeostasis and alleviated ER stress but also exerted dual regulatory effects on Aβ metabolism by inhibiting amyloidogenic enzyme expression while restoring the activity of Aβ-degrading proteases. These multifaceted actions set coixol apart from previously reported natural compounds with more limited mechanistic scope.

Despite these promising results, several limitations should be acknowledged. First, all experiments were conducted exclusively in a differentiated IMR-32 human neuronal cell model. Although this model expresses key AD–related proteins and responds robustly to CML-induced stress, it does not fully replicate the complexity of the in vivo brain environment, including glial–neuronal interactions, systemic pharmacokinetics, and blood–brain barrier dynamics. To address this limitation, future studies will incorporate in vivo animal models and/or human-derived brain organoids to validate the neuroprotective effects of coixol under physiologically relevant conditions and to evaluate its pharmacokinetic and safety profiles. Second, although commercially validated ELISA kits with high sensitivity and specificity were used, phosphorylation-specific targets such as *p*-PERK/PERK and *p*-eIF2α/eIF2α require confirmation by Western blotting in subsequent investigations. Third, the current study cannot definitively determine whether coixol acts directly on the PERK/eIF2α/ATF4/CHOP axis or indirectly via its antioxidant effects. To clarify this, future work will employ mechanistic approaches, including the use of specific PERK inhibitors, co-treatment with selective ROS scavengers, and targeted knockdown of PERK or ATF4, to distinguish direct from indirect actions on ER stress signaling pathways.

## 5. Conclusions

Our findings demonstrate that coixol effectively attenuates CML-induced neuronal damage by suppressing ER stress, mitigating apoptosis, and modulating both the production and clearance of Aβ. These neuroprotective actions underscore coixol’s ability to target multiple pathological processes implicated in AGEs/RAGE-mediated neurodegeneration. Given the central role of AGEs in the progression of metabolic-cognitive disorders, coixol emerges as a promising natural compound with disease-modifying potential, meriting further investigation in more physiologically relevant models.

## Data Availability

All the data needed to evaluate the conclusions in the paper are present in the paper. Additional data related to this paper may be requested from the authors.
